# Visual Cortical Processing in Children with Early Bilateral Cochlear Implants: A VEP Analysis

**DOI:** 10.3390/children12030278

**Published:** 2025-02-25

**Authors:** Ola Badarni-Zahalka, Ornella Dakwar-Kawar, Cahtia Adelman, Salma Khoury-Shoufani, Josef Attias

**Affiliations:** 1 Department of Communication, Sciences and Disorders, Haifa University, Haifa 3498838, Israel; obadar01@campus.haifa.ac.il; 2 School of Occupational Therapy, Hebrew University of Jerusalem, Jerusalem 9190501, Israel; ornella.dakwar@mail.huji.ac.il; 3 Speech & Hearing Department, Hebrew University-Hadassah Medical Center, Jerusalem 9112001, Israel; cahtiaa@hadassah.org.il (C.A.); salma@hadassah.org.il (S.K.-S.); 4 Department of Communication Disorder, Hadassah Academic College, Jerusalem 9101001, Israel

**Keywords:** cochlear implants, visual evoked potentials, cross-modal plasticity, speech perception, early intervention

## Abstract

Background/Objectives: Cochlear implantation is the primary treatment for severe-to-profound hearing loss, yet outcomes vary significantly among recipients. While visual–auditory cross-modal reorganization has been identified as a contributing factor to this variability, its impact in early-implanted children remains unclear. To address this knowledge gap, we investigated visual processing and its relationship with auditory outcomes in children who received early bilateral cochlear implants. Methods: To examine potential cross-modal reorganization, we recorded visual evoked potentials (VEPs) in response to pattern-reversal stimuli in 25 children with cochlear implants (CIs) (mean implantation age: 1.44 years) and 28 age-matched normal-hearing (NH) controls. Analysis focused on both the occipital region of interest (ROI: O1, OZ, and O2 electrode sites) and right temporal ROI, examining VEP components and their correlation with speech perception outcomes. Results: Unlike previous studies in later-implanted children, the overall occipital ROI showed no significant differences between groups. However, the left occipital electrode (O1) revealed reduced P1 amplitudes and delayed N1 latencies in CI users. Importantly, O1 N1 latency negatively correlated with speech-in-noise performance (r = −0.318; *p* = 0.02). The right temporal region showed no significant differences in VEP N1 between groups and no correlation with speech performance in CI users. Conclusions: Early bilateral cochlear implantation appears to preserve global visual processing, suggesting minimal maladaptive reorganization. However, subtle alterations in left occipital visual processing may influence auditory outcomes, highlighting the importance of early intervention and the complex nature of sensory integration in this population.

## 1. Introduction

While cochlear implants (CIs) have transformed the treatment of severe-to-profound hearing loss [1], significant variability in user outcomes persists, particularly in language acquisition and academic achievement. This disparity remains notable even when accounting for early intervention—typically around 3.5 years—and comprehensive rehabilitation programs [2,3,4].

A key factor in these varying outcomes is cross-modal reorganization following early auditory deprivation. During critical developmental periods, the auditory cortex adapts by processing input from other sensory modalities, particularly vision [5,6,7]. This neuroplastic response alters connectivity between auditory and non-auditory regions, potentially redirecting resources typically reserved for auditory development to support other sensory functions [8,9,10].

To better understand these adaptive processes and their implications for cochlear implant outcomes, researchers often employ visual evoked potentials (VEPs) as a valuable investigative tool. VEPs reveal important insights into sensory interactions and adaptations through three main components occurring at specific time intervals: P1 (t ≈ 100 ms), N1 (t ≈ 150–200 ms), and P2 (t ≈ 200–300 ms). The P1 component emerges around one month post-birth and functions as a marker of early sensory processing and visual cortex activation, showing responsiveness to both stimulus characteristics and initial attentional processes. This component is mainly found in striate and extrastriate areas, particularly involving the dorsal extrastriate cortex [11,12]. The N1 component, which follows P1, indicates more sophisticated perceptual processing and shows sensitivity to visual features and attention. It originates from various neural sources, with significant parietal involvement, and is especially active when tasks demand active visual discrimination [12,13,14,15]. Both the P1 and N1 components show age-related developmental changes, although quantifying N1 has proven challenging due to its complex waveform patterns [16,17]. The P2 component shows significant activation patterns in parieto-occipital regions, reflecting its role in visual sensory integration and attentional processes [18,19,20,21], and is crucial for perceptual closure and visual memory integration [22]. Most importantly, P2 serves as a critical neural marker that bridges the transition from basic visual sensory processing to sophisticated higher-order cognitive operations, highlighting its unique role in understanding cognitive processing hierarchies [18,23]. Together, these components create a temporal framework for understanding the stages of visual processing and their developmental progression.

The clinical significance of these VEP components becomes particularly relevant when considering their relationship to cochlear implant outcomes. Research has revealed a complex relationship between cross-modal reorganization and CI outcomes. While this neural plasticity can enhance compensatory mechanisms like visual speechreading in post-lingually deafened adults [24,25], increased visual system plasticity has also been linked to better post-implantation outcomes [26,27]. However, extensive auditory cortical reorganization can impede speech perception in CI users [28,29,30]. Although sensory system plasticity following deafness is not inherently maladaptive, its nature and extent can significantly impact CI rehabilitation success [10,31,32,33].

Building upon this understanding of neural plasticity’s dual nature, there is growing recognition of the need for individualized rehabilitation strategies, especially in pre-lingually deafened children. In these early stages, the absence of auditory input triggers substantial brain reorganization, fundamentally altering sound processing post-implantation [34]. This process, occurring during auditory deprivation, sees the auditory cortex undergo extensive restructuring, with visual processing systems often taking over traditionally auditory roles [6,7]. Two distinct forms of plasticity emerge: early plasticity, characterized by initial cortical reorganization during hearing loss, and late plasticity, reflecting post-implantation adaptations [23,35,36]. These critical developmental windows represent peak periods of neural plasticity [35]. Understanding cross-modal interactions is crucial for enhancing clinical outcomes in children with hearing loss. However, significant knowledge gaps persist regarding how cochlear implants (CIs) influence cross-modal plasticity in these children. This highlights the urgent need for further research to fully understand the mechanisms through which cochlear implantation aids in the recovery of hearing and speech functions [37]. Understanding these cross-modal interactions is vital for optimizing clinical outcomes. Nevertheless, significant knowledge gaps remain concerning the effects of CIs on cross-modal plasticity in children with hearing loss, underscoring the need for additional research to fully comprehend how cochlear implantation facilitates hearing and speech function recovery [37].

Research has consistently revealed significant differences in VEP components between children with CIs and those with NH, demonstrating the considerable impact of cross-modal reorganization on sensory and linguistic processing. This phenomenon is particularly evident in pre-lingually deaf children, as early auditory deprivation fundamentally reshapes neural architecture and subsequent auditory processing following implantation. In their seminal study, Campbell and Sharma (2016) [23] demonstrated that auditory processing regions, particularly the right temporal lobe, become recruited for visual processing in children with CIs. Their findings revealed that lower-performing CI users exhibited enhanced N1 amplitudes and shortened N1 latencies, which strongly correlated with difficulties in speech perception in noisy environments. These results suggest that cross-modal plasticity redirects auditory cortical resources toward visual processing, potentially compromising auditory function [23,38,39].

Further evidence of N1 component alterations in pediatric CI users has emerged from subsequent research. Liang et al. [40,41] documented increased N1 amplitudes, while both Corina et al. (2017) [42] and Campbell and Sharma (2016) [23] observed earlier N1 latencies. A recent study by Corina et al. (2024) [43] identified enhanced N1 negativity in CI users, along with distinct VEP patterns in children with congenital deafness that corresponded to increased visual attention but reduced spoken language performance. While these findings suggest that cross-modal plasticity may enhance visual processing capabilities, they also indicate potential interference with auditory development and language acquisition.

Source localization studies have further substantiated these observations. Campbell and Sharma (2016) [23] and Liang et al. (2017a, 2017b) [40,41] consistently demonstrated that morphological alterations in the N1 component were linked to increased activity in the right temporal lobe, particularly prominent among underperforming CI users. Recent functional near-infrared spectroscopy (fNIRS) studies by Zhao et al. (2022) [24] and Mushtaq et al. (2020) [44] have revealed heightened activation of auditory brain regions in response to auditory and visual speech stimuli in CI users positively associated with auditory speech comprehension abilities.

Studies examining visual processing differences have yielded additional insights. De Schonen et al. (2018) [45] and Gabr et al. (2022) [46] documented increased P1 amplitudes among CI users, suggesting enhanced visual processing capacities potentially attributable to cross-modal reorganization. However, Deroche et al. (2024) [36] observed that cross-modal reorganization in CI users, particularly those with language delays, often mani-fests maladaptively. Children with delayed language development demonstrated reduced visual cortex activity, diminished synchronization within auditory association areas, and the decoupling of auditory and visual functions. This disconnection, combined with an inability to suppress auditory-region activity during visual tasks, may impede linguistic development. Contrary to expectations, auditory deprivation did not enhance visual processing capabilities in children with poor language outcomes. These findings challenge the hypothesis of compensatory mechanisms, such as the use of visual speech to support language development in CI users, as proposed by Mushtaq et al. (2020) [44]. Notably, Deroche et al. (2024) [36] found that children with CIs with advanced language skills displayed no significant differences in VEP components compared to their normal-hearing peers, underscoring the variability in neural adaptations across individuals.

Together, these findings highlight the complex relationship between sensory deprivation, cross-modal plasticity, and auditory development in CI users. While some neural adaptations facilitate sensory integration, others may hinder auditory processing and language acquisition, emphasizing the need for early and targeted interventions to address these challenges in children with cochlear implants. Despite significant advances in research, critical gaps remain in our understanding of cross-modal reorganization and visual evoked potentials (VEPs) in pediatric CI users. Recent studies by Corina et al. (2024) [43] and Deroche et al. (2024) [36] have examined substantial groups of children with bilateral implants, underscoring the importance of early auditory intervention for sensory integration and language development. However, a deeper understanding of the developmental trajectories in young CI recipients is essential for developing effective targeted interventions. Our study builds on the recommendations from Campbell and Sharma (2016) [23] regarding the need for larger sample sizes (25 children with CIs vs. 14) and earlier implantation ages (1.44 vs. 3.6 years). We focus on examining neural adaptation patterns in children who receive early bilateral cochlear implants (CIs). Their research revealed distinct patterns in VEPs between CI users and NH children (age: 5–15 years), specifically in the occipital region of interest (ROI). Importantly, they discovered that an earlier latency of VEP N1 in the right temporal ROI was linked to poorer speech performance, indicating maladaptive cross-modal reorganization. This finding is highly relevant to our research, as we aim to explore how changes in visual processing among CI users might affect their speech perception performance. Supporting this, several studies have shown significant correlations between CI outcomes and intra-modal visual adaptations during visual tasks [29,36,45]. Together, these findings highlight the need to investigate the interplay between visual processing adaptations and speech outcomes in children with early bilateral CIs.

Our investigation focuses on the relationship between speech-in-noise perception and VEP components across visual and auditory regions in children receiving bilateral implants during the critical period for auditory-visual integration. We examine three primary hypotheses: (1) children with early bilateral implantation (≤1.5 years) will demonstrate VEP components comparable to those of normal-hearing peers, with differences being electrode-specific rather than global; (2) early bilateral implantation will minimize cross-modal reorganization, shown by similar VEP responses between CI and NH groups in temporal regions; and (3) speech-in-noise performance will be related to occipital VEP components. Through this comprehensive analysis, we aim to enhance the understanding of neural adaptation patterns and identify reliable predictors of speech perception success, ultimately contributing to the development of evidence-based rehabilitation protocols.

## 2. Materials and Methods

### 2.1. Participants

A total of 56 children were initially recruited through the Pediatric Cochlear Implant Program at Hadassah Medical Center (CI group) and public recruitment flyers (NH group) in Jerusalem, Israel. The strict inclusion criteria for the CI group required bilateral implantation before the age of 3.5 years, aided hearing thresholds between 20 and 30 dB with both implants, word recognition scores above 75% in quiet with bilateral implants, consistent device use, oral language communication, regular mainstream education placement, and medium-to-high socioeconomic status (based on maternal education). General exclusion criteria for all participants (CI and NH) included developmental disorders, neurological disorders, genetic syndromes, cognitive impairments, and non-native speaker status. For the CI group, additional exclusions included device failures and ear pathology. For the NH group, hearing thresholds above 20 dB were exclusionary. After applying these criteria (exclusion of three children with CIs—two due to technical issues and one due to cochlear malformation), the final sample included 53 participants (94.6% participation rate) aged 7.04–13.26 years (M = 9.56; SD = 1.86). A priori power analysis using G*Power (Version 3.1, Heinrich-Heine-Universität Düsseldorf, Düsseldorf, Germany [47]), based on maximal effect sizes reported in previous studies examining visual and auditory processing [43] in CI users, indicated that a minimum of 24 participants per group would provide 80% power to detect a medium effect size (f = 0.622) at α = 0.05 for between-group comparisons. Our final sample of 25 children with CIs and 28 NH children provided sufficient statistical power.

The groups were well matched across key demographic variables, showing no significant differences in age (CI: M = 9.79, SD = 1.81; NH: M = 9.19, SD = 1.8; t(51) = 1.24, *p* = 0.22), gender distribution (CI: 9F/16M; NH: 10F/18M; χ^2^(1) = 0.26, *p* = 0.610), native language distribution (CI: 7 Hebrew/18 Arabic; NH: 10 Hebrew/18 Arabic; χ^2^(1) = 0.32, *p* = 0.574), socioeconomic status (CI: 14 medium/11 high; NH: 12 medium/16 high; χ^2^(1) = 0.73, *p* = 0.392), and estimated IQ (CI: M = 119.00, SD = 25.21; NH: M = 125.00, SD = 21.94; t(51) = 0.93, *p* = 0.359). The estimated IQ was derived from the vocabulary and block design subtests of the WISC-IV [48], which provides a validated short-form IQ assessment [49].

Among the CI group (*n* = 25), twenty-two received simultaneous implantation, while three underwent sequential implantation with brief inter-implant intervals (0.49, 0.6, and 2.08 years). These relatively short intervals during peak auditory plasticity effectively represent “near-simultaneous” implantation, as supported by recent evidence showing comparable outcomes to simultaneous procedures [50,51,52].

The mean age at the first implantation was 1.44 years (SD = 0.72; range: 0.47–3.01), and at the second implantation was 1.57 years (SD = 0.724; range: 0.61–3.02). The distribution of cochlear implant systems across our cohort comprised Cochlear™ (Cochlear Ltd., Sydney, Australia; 72%, *n* = 18), MED-EL™ (MED-EL GmbH, Innsbruck, Austria; 24%, *n* = 6), and Advanced Bionics™ (Advanced Bionics LLC, Valencia, CA, USA; 4%, *n* = 1), with all CI recipients achieving aided thresholds of 20–30 dB HL across frequencies. While the manufacturer distribution was uneven, the recent literature, including a 2023 scoping review [53] and earlier work by Harris and colleagues (2011) [54], has indicated no significant performance differences between current-generation CI systems regarding speech outcomes.

### 2.2. Study Procedures

The current study received approval from the Institutional Review Board (IRB) of Hadassah Hebrew Medical Center in Jerusalem. The current study was approved by the Institutional Review Board (IRB) of Hadassah—Hebrew Medical Center in Jerusalem, Israel (approval # HMO-0881-20), and written informed consent was obtained from all parents or guardians of the participants. The entire procedure lasted approximately three hours, with breaks provided at the participants’ request. Families received a compensation of USD 40 for their time and effort.

The entire procedure lasted approximately three hours, including breaks at the participants’ request. Families were compensated with USD 40 for their time and effort. Upon arrival, all participants were given a detailed explanation of the experimental tasks. Demographic and background data were collected through a questionnaire. For the NH group, pure-tone audiometric thresholds were assessed to confirm normal hearing levels in both ears.

#### Speech Perception Measures

To assess the perception of spoken sentences, either the Hebrew or the Arabic everyday sentences test for children was utilized (“MELE Sentences”) [55,56]. This test comprises 11 lists of 12 sentences each, selected based on criteria from the Central Institute of the Deaf (CID) [57]. The sentences utilize common words, include statements and questions, cover concrete topics, avoid private names, and vary in length from two to six words. Each list contains yes/no questions, declarative phrases, and imperative sentences. The sentences were recorded by a female native speaker, ensuring authenticity.

The testing environment simulated challenging listening conditions with a signal-to-noise ratio (SNR) of 0, chosen to avoid floor and ceiling effects based on pilot data from five children with CIs and five NH peers. For the everyday sentences test, scoring was based on the number of correct words in each sentence. Each correct word was awarded 2 points, while definitive morphemes in Hebrew/Arabic received 1 point.

Testing was conducted individually in a soundproof room, with participants seated one meter from a loud speaker at 0 degrees azimuth. Sentences-in-noise tests were delivered via an Asus laptop connected to the monitor view meter of a GSI audiometer. To ensure precision, sentence intensity was calibrated to 65 dB SPL at the participant’s location using a sound-level meter and a 1000 Hz calibration tone.

### 2.3. EEG Recording

Task and Stimuli: Participants were seated one meter from the laptop screen and instructed to remain as still as possible to minimize motion-related artifacts. An experimenter was present to help maintain the children’s attention on the task. Throughout the experiment, the children were asked to focus on a black dot positioned at the center of the pattern.

All participants were shown a high-contrast sinusoidal concentric grating that smoothly transitioned into a radially modulated grating, forming a circle–star pattern (Supplementary Materials, Figure S1). In each block, the circle and star patterns were displayed 150 times. The star grating was presented for 600 ms, immediately followed by the circle grating for another 600 ms. This morphing effect between the circle and star patterns created an illusion of motion and shape transformation, theoretically engaging both the dorsal (“where”) and ventral (“what”) visual pathways [18,21].

The experiment consisted of two blocks, each containing 300 stimulus presentations, resulting in a total recording time of three minutes per block. The VEP was time-locked to the onset of each star and circle grating. The protocol was implemented using PsychoPy (https://www.psychopy.org/, (accessed on 20 February 2025)).The protocol was implemented using PsychoPy version 2021 (University of Nottingham, Nottingham, UK; https://www.psychopy.org/, (accessed on 20 February 2025)).

#### 2.3.1. Data Recording and Analysis

A high-density 32-electrode sensor array net was placed on the scalp using the Micromed™ SD 64 system (Mogliano Veneto, Italy), following the 10–20 system (Spes Medica™, Battipaglia, Italy). Data were collected from 32 scalp locations: Fp1, Fp2, F7, F3, Fz, F4, F8, FT7, FC3, FCz, FC4, FT8, T7, C3, Cz, C4, T8, TP7, CP3, CPz, CP4, TP8, P7, P3, Pz, P4, P8, O1, Oz, and O2. The right earlobe (A2) was used as a reference, while the left earlobe (A1) served as the ground. The impedance was maintained below 10 kΩ throughout the recording. Raw data were sampled at a rate of 1024 Hz (EGI net amps 300 system) and stored for offline analysis.

#### 2.3.2. Preprocessing

EEG data analysis was conducted using custom MATLAB (2022) version R2022b (Natick, MA, USA: The MathWorks Inc; available at https://www.mathworks.com/products/matlab.html (accessed on 20 February 2025)) scripts in conjunction with EEGLAB (version 14.0.0) [58].

EEG data analysis was conducted using custom MATLAB scripts (version R2022b, MathWorks Inc., Natick, MA, USA; available at MathWorks, accessed on 20 February 2025) in conjunction with EEGLAB (version 14.0.0, Swartz Center for Computational Neuroscience, University of California San Diego, San Diego, CA, USA) [58].

 During the preprocessing phase, channels located near the speech processor and transmitter coil (Tp7, P7, TP8, P8) were excluded, following the same protocol applied to normal-hearing (NH) participants. Continuous EEG data were down-sampled to 250 Hz and re-referenced offline to the average of 26 electrodes. Subsequently, a finite impulse response (FIR) bandpass filter was applied, set between 0.1 and 30 Hz, while preserving the original sampling rate. Following recommendations by Widmann et al. (2015), FIR filtering was chosen for its zero-phase distortion capability and guaranteed stability, ensuring optimal signal preservation [36,59,60]. This meticulous preprocessing ensured the integrity of the data, facilitating subsequent analyses.

Next, artifacts were identified in a semi-automated manner, with sections of data exceeding 200 μV being automatically removed. Then, a visual inspection was made to identify and exclude sections of EEG traces containing substantial artifacts. Independent components associated with ocular and muscular artifacts were identified using the runica algorithm (via the pop_runica function), informed by visual inspection of scalp topography, power spectra, and time courses [61]. On average, 1.1 ± 0.02 eye-related components were removed, facilitating further analysis of the remaining components. The sampling window from which data were then epoched was from −100 to 500 ms relative to sound onset, with baseline correction applied from −100 to 0 ms. The data were epoched within a time window from −100 ms to 500 ms relative to the onset of the sound. A baseline correction was applied during the interval from −100 ms to 0 ms, ensuring an accurate analysis of the auditory event-related potentials (ERPs). To mitigate electrical artifacts from the cochlear implant (CI), we performed an independent component analysis (ICA) using the infomax algorithm on the epoched sampled data, utilizing the ADJUST plugin [62,63,64]. CI artifact components, which are several orders of magnitude larger than neural signals, present as systematically occurring amplitude peaks that are independent of brain processes [64,65,66]. These artifacts were identified based on three key features: the localized topographical distribution near the implant site, periodic patterns synchronized with device stimulation, and high-amplitude electrical transients [66,67,68,69] On average, a total of 0–2 ICA components were removed during this process. On average, a total of 0–2 ICA components were removed during the artifact correction process.

Noisy channels were spherically interpolated, averaging 1.98 electrodes (SEM: 0.28; range: 0–3 electrodes). Further artifact rejection was conducted automatically, excluding trials with voltages exceeding ±120 μV using ERPLAB. After completing all artifact rejection steps and excluding these trials, we found no significant differences between groups in terms of the number of accepted trials for ERP analysis (CI: M = 94.29, SE = 0.93% vs. NH: M = 93.69, SE = 0.85%, t(51) = 0.48, *p* = 0.635).

#### 2.3.3. EEG Data Analysis

Single-subject visual evoked potentials (VEPs) were averaged for each participant at electrodes in the occipital region (O1, O2, and Oz) using the ERPLAB toolbox, an open-source plugin for EEGLAB [58,66]. The amplitude and latency of VEP peaks for the averaged responses were measured at each individual electrode and in a region of interest (ROI), defined as the average of the three occipital electrodes. VEP measurements were also taken on right temporal ROIs (T8 and FT8).

VEP amplitude measurements were recorded as follows: the P1 amplitude was measured from the P1 peak to the N1 peak, and the N1 amplitude from the N1 peak to the P2 peak [23,70,71]. The peak latency was defined as the midpoint of the peak. Individual waveforms were averaged together to create a grand-average waveform for each group (NH and CI). To prepare the group VEP waveforms for presentation, they were low-pass filtered offline at 30 Hz. The peak detection time windows were determined through visual inspection of the grand-average event-related potential (ERP) waveforms. The P1 component was observed in the 100–200 ms time window, while the N1 component was observed in the 200–350 ms time window.

### 2.4. Statistic Analysis

Data analysis was conducted using IBM SPSS Statistics version 29 (IBM Corp., Armonk, NY, USA). In the data preprocessing phase, outliers were identified using the Interquartile Range (IQR) method [72]. Specifically, values were considered outliers if they fell outside the range [Q1 − 1.5 × IQR, Q3 + 1.5 × IQR], where Q1 and Q3 are the first and third quartiles, respectively, and IQR is the difference between Q3 and Q1. This robust statistical approach helped identify observations that deviated significantly from the overall pattern of the data. The prevalence of outliers across variables remained 1% for both CI and NH groups, with missing data rates of less than 2% for the NH and below 1% for the CI groups.

Normality assessment using the Shapiro–Wilk test and Q-Q plots revealed that most neural and demographic measures exhibited normal distribution (*p* > 0.05) for both groups. However, exceptions were noted in 4 out of 16 neural measures, 1 out of 3 demographic measures, and the speech-in-noise test scores. To address these non-normal distributions, various transformation methods were applied, including log_10 and Yeo–Johnson transformations with different lambda values. While parametric tests were subsequently employed for analyzing correlations between behavioral and neural measures, the speech-in-noise test data remained non-normalized despite transformation attempts.

For group comparisons, demographic characteristics (age, sex, and estimated IQ) were analyzed using independent *t*-tests and chi-square tests. Behavioral outcomes, specifically speech-in-noise performance, were compared using the Mann–Whitney U test. VEP responses between groups were evaluated using *t*-tests to assess differences in amplitude and latency within the occipital region of interest (ROI). A repeated measures ANOVA was implemented to examine the effects of the group (CI vs. NH) and electrode position (O1, O2, Oz) on each VEP response component (P1 amplitude, P1 latency, N1 amplitude, N1 latency). This model incorporated the effects of the electrode on the within-subjects and electrode–group interaction and the between-subjects effects of the group. Significant differences between CI and NH groups at each electrode were further investigated through post hoc comparisons.

The relationship between variables was examined using correlation analyses. Pearson correlations were employed to assess associations between demographic measures and VEP components, while Spearman correlations were used to investigate the relationship between behavioral outcomes (speech-in-noise test) and VEP components in both occipital and temporal ROIs. This comprehensive analytical approach enabled a thorough understanding of how auditory intervention influences visual processing in children with cochlear implants.

## 3. Results

### 3.1. Group Differences in VEP Components in the Occipital ROI

The analysis of Visual Evoked Potentials (VEPs) EPs indicated that the latency and amplitudes of the VEP components of the CI users are similar to those of the NH children within the occipital region of interest (ROI), with no statistical differences (Supplementary Materials, Figure S1). Independent two-sample *t*-tests were conducted on the standardized scores for both amplitude and latency measures of the P1 and N1 components.

**Figure 1:**
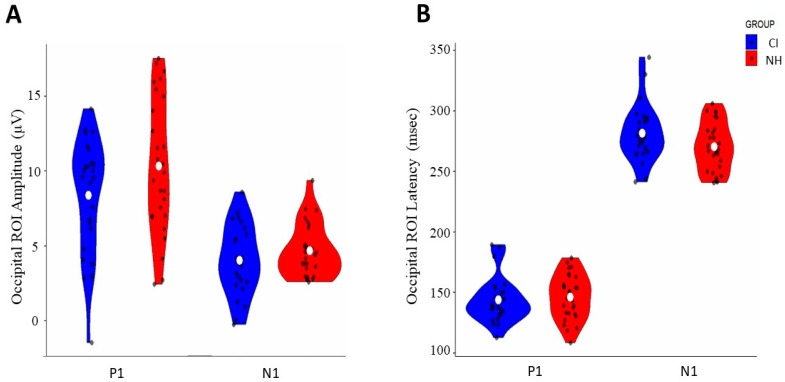
Comparison of occipital ROI VEP responses between CI and normal-hearing (NH) groups: (**A**) amplitude distribution of P1 and N1 components (μV) and (**B**) latency distribution of P1 and N1 components (ms). Blue violin plots represent the CI group, and red violin plots represent the NH group. White dots indicate mean values, and black dots show individual data points.

The comparison of VEP peak components between CI and NH groups using independent *t*-tests revealed no significant differences in amplitude or latency measures. For amplitude measures (Figure 1A, Table 1), neither the P1 component [t(50) = −1.73, *p* = 0.089, d = −0.47] nor the N1 component [t(50) = −1.21, *p* = 0.231, d = −0.309] showed significant group differences. Similarly, latency analysis (Figure 1B, Table 1) demonstrated no significant differences between groups for either P1 [t(51) = −0.45, *p* = 0.657, d = −0.12] or N1 components [t(51) = −1.832, *p* = 0.073 d = 0.51].

The analysis of visual evoked potentials (VEPs) indicated that visual cortex processing is comparable between children with cochlear implants (CIs) and those with normal hearing (NH) within the occipital region of interest (ROI). Independent two-sample *t*-tests were conducted on the standardized scores for both amplitude and latency measures of the P1 and N1 components.

### 3.2. Group-Specific Variations in Visual Evoked Potential Components Across Occipital Electrode Sites

**Figure 2:**
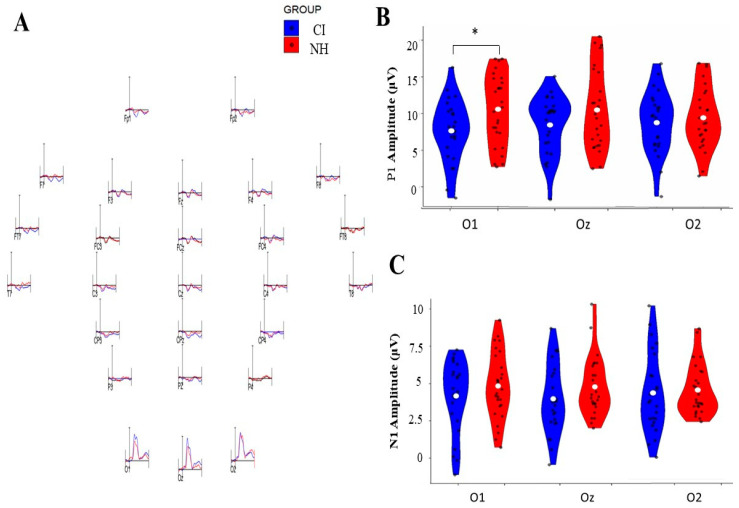
Comparison of P1 and N1 VEP amplitudes between cochlear implant (CI) and normal-hearing (NH) groups across three occipital electrode sites (O1, Oz, O2). (**A**) Representative VEP waveforms were recorded from different electrode positions for the CI and NH groups. (**B**) Violin plots showing P1 amplitude distributions (0–20 μV) for both groups, with significant differences (* *p* < 0.05) observed at O1. (**C**) Violin plots showing N1 amplitude distributions (0–10 μV) for both groups across the three occipital sites. Blue plots represent CI group data, while red plots represent NH group data. White dots indicate mean values, and black dots show individual measurements.

**Figure 3:**
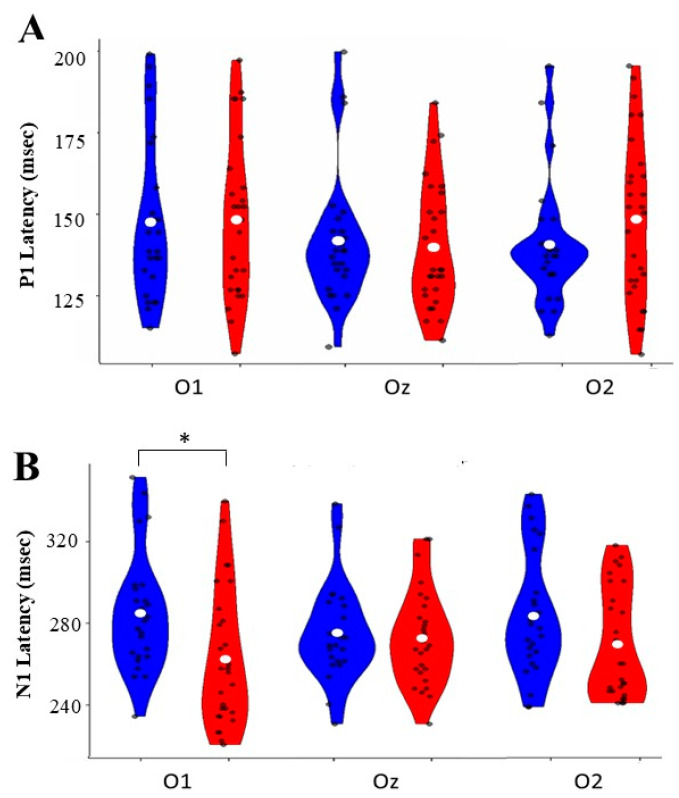
Normal-hearing (NH) groups at three occipital electrode sites (O1, Oz, O2). (**A**) P1 latency distributions show ranges between 125 and 200 ms across all sites. (**B**) N1 latency distributions ranged from 240 to 320 ms, with a significant difference (* *p* < 0.05) between groups at O1. Blue violin plots represent CI group data, and red plots represent NH group data. White dots indicate mean values, and black dots show individual measurements.

Hemispheric asymmetries were assessed by analyzing the VEP components, revealing distinct hemispheric asymmetries between groups across the occipital electrode positions (O1, Oz, and O2). Notable differences between groups emerged at the left hemispheric O1 electrode site [F (1, 50) = 5.378, *p* = 0.025, η^2^*p* = 0.097]. We analyzed their amplitude and latency parameters by analyzing both positive and negative early components of VEP, specifically the P1 and N1 components. The results are detailed in Table 2 and Table 3 and illustrated in Figure 2 and Figure 3.

#### 3.2.1. Amplitude Analysis

P1 Component: The analysis revealed no significant main effects for electrode position [F (2, 48) = 0.157, *p* = 0.855, η^2^*p* = 0.006], group membership [F (1, 48) = 2.056, *p* = 0.158, η^2^*p* = 0.040], or their interaction [F (2, 48) = 2.681, *p* = 0.079, η^2^*p* = 0.100]. However, pairwise comparison analysis revealed a significant group difference, specifically at the O1 electrode, where the CI group demonstrated a reduced amplitude compared to the NH group [mean difference = −0.572; *p* = 0.035; 95% Confidence Interval [−1.104, −0.041]]. No such differences were observed at O2 [mean difference = −0.086, *p* = 0.755, 95% CI [0.640 to 0.467]] or Oz [mean difference = −0.422, *p* = 0.133, 95% Confidence Interval [0.978, 0.134]].

N1 Component: The N1 amplitude analysis showed no significant main effects for the electrode [F (2, 50) = 0.001, *p* = 0.999, η^2^*p* = 0.000], group [F (1, 50) = 1.744, *p* = 0.193, η^2^*p* = 0.034], or their interaction [F (2, 50) = 0.483, *p* = 0.618, η^2^*p* = 0.010]. Pairwise comparisons confirmed the absence of significant differences between groups across all electrode sites: O1 [mean difference = −0.290, *p* = 0.301, 95% Confidence Interval [−0.847, 0.267]], O2 [mean difference = −0.269, *p* = 0.338, 95% Confidence Interval [−0.827, 0.289]], and Oz [mean difference = −0.422, *p* = 0.112, 95% Confidence Interval [0.991, 0.106]].

#### 3.2.2. Latency Analysis

P1 Component: P1 latency analyses revealed no significant main effects for the electrode [F (2, 50) = 0.004, *p* = 0.996, η^2^*p* = 0.000], group [F (1, 50) = 0.086, *p* = 0.771, η^2^*p* = 0.002], or their interaction [F (2, 50) = 1.236, *p* = 0.295, η^2^*p* = 0.024]. Pairwise comparisons showed no significant differences between groups at any electrode site: O1 [mean difference = −0.062, *p* = 0.916, 95% CI [−0.616 to 0.742]], O2 [mean difference = 0.255, *p* = 0.308, 95% Confidence Interval [−0.255 to 0.725]], or Oz [mean difference = 0.097, *p* = 0.705, 95% Confidence Interval [−0.401 to 0.546]].

N1 Component: While the analysis showed no significant main effects for the electrode [F(2, 49) = 0.020, *p* = 0.965, η^2^*p* = 0.001] or group [F(1, 50) = 0.590, *p* = 0.446, η^2^*p* = 0.012], there was a significant interaction [F(2, 49) = 4.169, *p* = 0.021, η^2^*p* = 0.146]. Pairwise comparisons revealed a significant difference at the O1 electrode [mean difference = −0.590, *p* = 0.025, 95% Confidence Interval [0.079, 1.101]]. The CI group demonstrated shorter latency at this site than the NH group. No significant differences were observed at O2 [mean difference = −0.414, *p* = 0.129, 95% Confidence Interval [−0.954, 0.125]] or Oz [mean].

### 3.3. Speech-in-Noise Performance Between Groups

**Figure 4:**
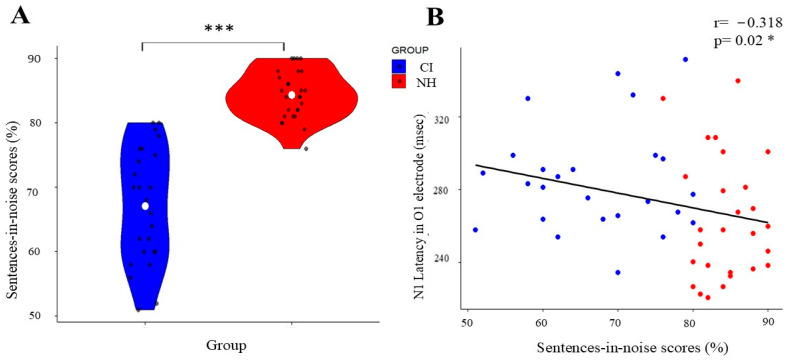
Correlation between speech perception and N1 NEP latency in CI and NH groups. (**A**) Violin plot showing the distribution of sentences-in-noise scores (%), with the CI group in blue and the NH group in red. White dots indicate mean values; black dots show individual data points. (**B**) Scatter plot demonstrating the negative correlation (r = −0.318, *p* = 0.02 *) between sentences-in-noise scores and N1 latency in the O1 electrode across both groups, with regression line showing the inverse relationship. (*) indicates significant differences between the groups (* *p* < 0.05, *** *p* < 0.001).

The analysis of speech-in-noise performance revealed significant differences between the groups. Normal-hearing (NH) participants demonstrated significantly superior performance (mean rank = 39.16) compared to the cochlear implant (CI) group (mean rank = 13.38; Mann–Whitney U = 9.500, *p* < 0.001). As shown in Figure 4A, the NH group consistently achieved higher scores in the sentences-in-noise task.

### 3.4. VEP Components and Speech-in-Noise Correlations

While we did not observe the expected temporal lobe differences, our analysis revealed an interesting relationship in occipital regions. Specifically, the Spearman’s rank correlation analysis of the unstandardized VEP measurements demonstrated a significant relationship between visual processing and speech perception abilities, with N1 latency at the O1 electrode showing a moderate negative correlation with sentences-in-noise performance (rs = −0.318, *p* = 0.02; Figure 4B).

### 3.5. Group Differences in VEP Components in the Right Temporal ROI

We found no significant differences in N1 latency in the right temporal ROI between groups [t(51) = 0.93, *p* = 0.35; Confidence Interval 95% [−5.16, 14.15], d = 0.257]. In addition, this measure had no correlation with speech-in-noise performance (rs = −0.147, *p* = 0.294).

### 3.6. Age-Related Changes in Visual Processing

**Figure 5:**
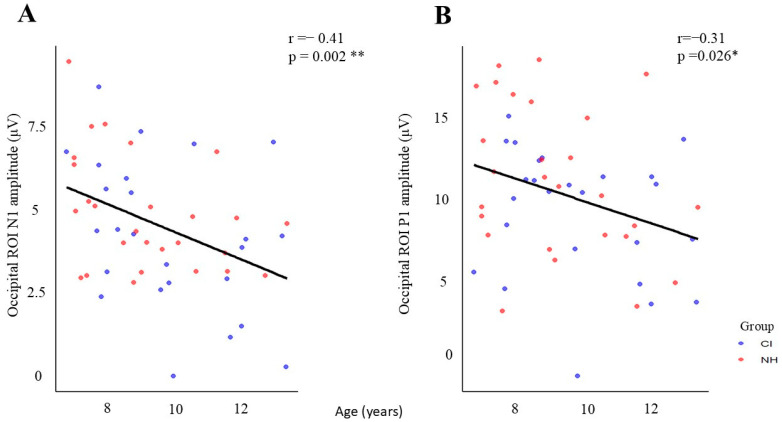
Age-related changes in occipital ROI amplitudes for CI and NH groups. (**A**) N1 amplitude shows a significant negative correlation with age (r = −0.41, *p* = 0.002 **), and (**B**) P1 amplitude also demonstrates a significant negative correlation with age (r = −0.31, *p* = 0.026 *). Blue dots represent CI participants, and red dots represent NH participants. Black regression lines indicate the overall trend across both groups (* *p* < 0.05, ** *p* < 0.01).

An analysis of demographic correlations revealed significant age-dependent changes in visual processing across both groups. Two notable correlations were identified: the P1 amplitude showed a moderate negative correlation with age across the occipital region of interest (ROI) (r = −0.309, *p* < 0.05), while the N1 amplitude exhibited a stronger negative correlation with age (r = −0.411, *p* < 0.01). This indicates that both P1 and N1 amplitudes decrease as chronological age increases. These correlations demonstrate that both children with cochlear implants (CIs) and those with normal hearing (NH) exhibit reduced response amplitudes in both early (P1) and later (N1) stages of visual processing as they age (Figure 5), consistent with typical neurodevelopmental trajectories.

Neither implant age nor the duration of implant use showed significant correlations with any VEP components (see Supplementary Table S2).

## 4. Discussion

Our findings reveal distinct visual processing patterns in children with early bilateral cochlear implants. The results demonstrate how early auditory intervention may help maintain typical-like visual processing patterns in the occipital region. Notably, we observed a key difference at the left occipital electrode (O1), where CI recipients showed reduced P1 amplitude and delayed N1 latency compared to NH peers. These findings provide valuable insights into neural plasticity in early-implanted children and have important clinical implications, which we discuss in detail below.

### 4.1. Preserved Global Visual Processing

Our investigation found no significant differences in visual evoked potential (VEP) responses within the occipital region of interest (ROI) between children with CI and those with NH. This aligns with the findings of Deroche et al. (2024) [36], who also reported no statistically significant differences in occipital activation between NH controls and (high-language) CI users who demonstrated high language abilities scores above average (>100), as measured by the Clinical Evaluation of Language Fundamentals (CELF). Interestingly, our results indicate typical visual processing in early-implanted CI users, contrasting with the earlier literature documenting enhanced visual responses and shortened latencies as markers of compensatory cross-modal plasticity [23,46]. The differences between our findings and those of previous studies can be attributed to several key advancements in methodology.

Most notably, our study involved a significantly larger cohort (*n* = 25) compared to Campbell and Sharma (2016) [23] (*n* = 14). Furthermore, our participants underwent bilateral implantation much earlier (mean = 1.5 years) than those in their study (mean = 3.5 years). Additionally, while Gabr et al. (2022) [46] focused on unilateral CI users with limited auditory outcomes (mean word recognition score (WRS) = 54.46%), our early-implanted bilateral CI users exhibited good auditory performance in quiet conditions (WRS = 88%).

The preservation of nearly typical visual processing in our cohort demonstrates the advantages of early bilateral implantation, which ensures balanced auditory input during critical developmental periods. The developing brain can maintain typical sensory processing patterns when auditory input is restored early through bilateral implantation during sensitive developmental windows [32,34]. This early intervention helps prevent the substantial neural reorganization typically observed with prolonged auditory deprivation [38,72]. The timing of intervention appears crucial, as it allows the auditory system to develop while maintaining the integrity of other sensory processing networks and either mitigates or reverses cross-modal reorganization.

### 4.2. Cross-Modal Reorganization and Temporal Processing

The current study shows different indications of cross-modal reorganization observed in previous studies, specifically in Campbell and Sharma (2016) [23]. Contrary to previous findings, we did not find a significant difference in N1 latency in the temporal ROI between CI and NH peers. This discrepancy may be attributed to our cohort’s early bilateral CI intervention and extended period of CI use, which also preserved overall occipital ROI functioning. Indeed, we did not find any relationship between temporal N1 latency and speech outcomes. This contrasts with Campbell and Sharma (2016) [23], who reported that shorter latencies in temporal lobe N1 were associated with poorer speech perception in noise in their CI cohort.

The timely implementation of cochlear implants in pre-lingually deaf children leads to distinctive auditory–visual cortical patterns. Over time, prolonged implantation triggers a reversal of cross-modal functional changes in the brain, gradually restoring typical activity [37,38].

The lack of differences in N1 responses and the absence of association with speech perception in noise suggest that cross-modal reorganization is absent in this temporal region among our children with CIs [23]. Recent studies support this finding, demonstrating that cross-modal reorganization due to auditory deprivation in pre-lingual CI children (age 2–7 years) is transient after auditory reactivation, with reorganization observed temporarily at 3 months post-surgery before returning to typical levels (compared with the control group) by 12 months post-surgery [37].

Consequently, while cross-modal reorganization may not be evident in temporal areas, it is worth investigating whether visual processing characteristics in occipital regions play a role in speech perception.

### 4.3. Localized Adaptations in Visual Processing and Association with Speech Perception in Noise

By analyzing VEP responses at each site, our investigation revealed a distinctive pattern of neural adaptation in CI users that challenges the previous understanding of compensatory mechanisms in sensory processing. While global visual processing remained intact, we observed specific alterations at the left lateral site (O1), characterized by reduced P1 amplitudes and delayed N1 latencies. These hemisphere-specific findings present an intriguing contrast to the traditionally reported pattern of enhanced visual responses across occipital regions, which has been extensively documented in deaf animals [73,74,75], post-lingually deaf adults [28,29], and pediatric CI populations [23,40,41,45,46]. The established literature consistently demonstrates increased P1 amplitudes [45,46] and shorter N1 latencies [23,41], suggesting a general enhancement of visual processing capabilities in individuals with hearing loss.

Our findings, however, align more closely with recent work by Deroche et al. (2024) [36], who reported decreased P1 amplitudes in their CI users. However, there are important distinctions between the studies. Deroche and colleagues observed a reduced P1 amplitude across all occipital regions of interest (ROIs), specifically in their low-language CI group. In contrast, the reduced P1 amplitude in our cohort was identified only at the left occipital (O1) electrode site, while the overall P1 response in the broader occipital ROI was preserved. This suggests that the observed reduction in P1 amplitude in our CI users may be more localized to the left occipital area, rather than a global effect across the entire occipital cortex

Yet, important distinctions exist between these studies. Deroche and colleagues observed a reduced P1 amplitude across all occipital regions of interest (ROIs), specifically in their low-language CI group [32]. This finding was identified in our cohort just at the left O1 electrode site, although the overall P1 occipital ROI was preserved. 

This difference might be attributed to our cohort’s language abilities—while we did not directly assess language performance, our participants with CIs showed no significant differences in estimated IQ (which included vocabulary subtests) compared to NH controls. The fact that alterations appeared exclusively in the left hemisphere suggests more nuanced neural adaptations among children with CIs who were implanted early. The preserved P1 responses at other electrode sites might reflect successful neural recovery following early bilateral implantation in children with good language outcomes, while the persistent left hemisphere alterations suggest that some neural modifications remain present. These lasting left-lateralized adaptations could potentially influence CI outcomes and warrant further investigation.

On further exploration of these hemispheric patterns and their functional implications, the described alteration in the left hemisphere was found to correlate with speech perception in noise, demonstrating a significant negative correlation between left hemisphere visual processing efficiency (measured through N1 latencies) and speech-in-noise performance (rs = −0.318, *p* = 0.02). These findings support De Schonen et al.’s (2018) [45] hypothesis that larger or longer VEP responses may hinder speech perception. Their longitudinal study of pre-lingual deaf infants (*n* = 25, mean age = 39 months, SD = 16.8) revealed that enhanced visual VEP N1 amplitude asymmetry (quantified as left minus right hemisphere activation) predicted poorer speech perception outcomes three years post-implantation. Additional evidence from Sandmann et al. (2012) [29], although not directly examining hemispheric asymmetry, reinforces this relationship by demonstrating that enhanced P100 responses to visual stimulation in post-lingual deaf adults with CIs were inversely associated with speech outcomes. These convergent findings indicate that elevated visual processing, particularly within the left hemisphere, may signal suboptimal conditions for speech perception in CI users.

Interestingly, the association between speech-in-noise performance and visual processing efficiency observed in our study was also present in children with normal hearing (NH), indicating that this relationship extends beyond the CI population. The parallel between CI users and NH individuals raises important questions about the nature of left hemisphere specialization and its role in language processing. The left hemisphere’s specialization for speech processing appears to be an inherent feature rather than a compensatory mechanism. This is supported by observations of similar patterns in normal-hearing individuals during speech-processing tasks [76,77]. Furthermore, the correlation between visual processing measures and speech-in-noise performance in the left hemisphere suggests the involvement of shared neural resources or common processing mechanisms. This highlights the fundamental role of the left hemisphere in language processing, regardless of the sensory input modality [78,79].

### 4.4. Developmental Trajectory of VEP Amplitudes

Analyzing demographic factors revealed significant age-related changes in visual processing among children with CIs and those with NH. As chronological age increases, both the P1 and N1 amplitudes decrease. These correlations indicate that children with cochlear implants and those with normal hearing both demonstrate reduced response amplitudes in the early (P1) and later (N1) stages of visual processing as they age. This pattern is consistent with typical neurodevelopmental trajectories [16,17].

Our findings align with those of Corina et al. (2024) [43], which compared 28 children with cochlear implants (mean age = 74 months; range: 46–128 months) to 28 age-matched controls (mean age = 78 months; range: 31–122 months). The study found that increasing age is associated with reduced amplitude and shorter latencies in visual evoked potentials (VEPs). This suggests that age-related modifications in VEPs reflect increased neural efficiency and specialization. Additionally, a comprehensive perspective provided by Deroche et al. reveals that developmental changes in VEPs are influenced by factors such as variable myelination across brain regions, the region-specific timing of dendritic branching, and increased skull size and thickness due to head growth. All these factors contribute to reducing VEP amplitudes in older children. Therefore, to interpret changes in VEPs accurately, it is crucial to adopt a comprehensive approach that considers both the functional maturation of neural responses and the physical and anatomical development of the brain [80,81].

Importantly, neither the age of the implant nor the length of time it had been used showed any significant correlations with visual evoked potential (VEP) components. This lack of correlation suggests that the timing of cochlear implantation may not greatly affect visual processing characteristics, especially since the CI group received their first implants at a young age. These findings indicate that while visual processing undergoes typical age-related changes in both groups, these changes seem to be driven by natural developmental processes rather than factors related to the implants.

## 5. Conclusions

Our study reveals three key findings that advance our understanding of sensory processing in early-implanted children. First, we found preserved typical global visual processing in the occipital region, challenging previous assumptions about inevitable cross-modal reorganization in deaf children. Second, we identified specific adaptations in the left occipital region (O1), characterized by reduced P1 amplitude and delayed N1 latency. Third, we discovered a significant correlation between visual processing efficiency and speech-in-noise performance, which extends to normal-hearing children.

These findings strongly support the benefits of early bilateral cochlear implantation in maintaining typical sensory processing patterns. The preservation of typical visual processing suggests that the timing of intervention is crucial for optimal sensory development. The correlation between visual processing and speech performance offers a potential new tool for predicting rehabilitation outcomes.

Our results have immediate clinical implications, supporting early identification and intervention in congenital hearing loss and providing a theoretical framework for understanding sensitive periods in sensory development. 

Future research should address the long-term stability of these adaptations and their relationship to academic and social outcomes. Longitudinal studies could explore how visual processing patterns evolve throughout development and their continued impact on auditory performance.

## 6. Limitations

When interpreting our findings, several limitations should be considered. Despite our efforts to maintain a homogeneous CI cohort (*n* = 25), some heterogeneity was present in the implantation approach, with 22 individuals receiving simultaneous implants and 3 receiving sequential implants. Notably, the sequential cases had relatively short inter-implant intervals (two children at 0.6 years and one at 2.08 years), which could be considered quasi-simultaneous compared to typical sequential intervals in the literature. This might explain their similar patterns to the simultaneous group, though this requires further investigation.

Regarding CI devices, our study included participants with various device configurations and speech processors, which may have introduced uncontrolled variables. While the majority (88%) had bilateral simultaneous implants from a single manufacturer, future studies should consider systematic comparisons between different CI manufacturers (e.g., Cochlear, Advanced Bionics, MED-EL) to evaluate whether device-specific characteristics and processing strategies differentially affect visual system plasticity and cross-modal reorganization.

The duration of implant use varied (6–12.77 years), and our restricted age range (7–13 years) may affect the result in generalizability. Our single-session, cross-sectional format limits our understanding of developmental trajectories. A longitudinal approach would be particularly valuable to (1) track visual processing changes from pre- to post-implantation, (2) examine how bilateral implantation timing affects cross-modal plasticity during critical developmental periods, (3) monitor the relationship between auditory rehabilitation and visual processing changes, and (4) assess whether the similarities between quasi-simultaneous and simultaneous implantation persist over time.

Technical limitations included using a 32-channel EEG system and potential residual CI artifacts. The speech-in-noise testing at a single SNR level and limited cognitive assessments (two WISC-IV subtests) may not fully reflect participants’ abilities. Future research should address these limitations through longitudinal designs, larger samples, multiple visual paradigms, comprehensive assessments, and higher-density EEG recordings.

## Figures and Tables

**Table 1:** Means and SDs (standard deviations) for the VEP components (P1 and N1 amplitudes and latencies) for cochlear implant (CI) users and normal-hearing (NH) individuals, along with the results of independent-samples *t*-tests.

VEP Component	CI (*n* = 25) Mean (SD)	NH (*n* = 28) Mean (SD)	t Value(df)	*p*-Value	95% CI	Effect Size (Cohen d)
P1 Amplitude (μV)	8.35 (3.86)	10.41 (4.61)	−1.73 (50)	0.089	[−4.309, 0.369]	−0.47
N1 Amplitude (μV)	4.04 (2.32)	4.76 (1.95)	−1.21 (50)	0.231	[−1.77, 0.509]	−0.309
P1 Latency (ms)	143.00 (18.99)	146.00 (18.45)	−0.45 (51)	0.657	[−12.63, 8.03]	−0.12
N1 Latency (ms)	284.27 (26.96)	270.35 (20.06)	−1.832 (51)	0.3	[−0.994, 23.509]	0.513

**Table 2:** Comparison of P1 and N1 amplitude between cochlear implant (CI) and normal-hearing (NH) groups: results of repeated measures ANOVA including main effects, interaction, and post hoc analyses across electrode sites (O1, O2, and Oz).

Amplitude (μV)	Effect Type	Source	CI (*n* = 25) Mean (SD)	NH (*n* = 28) Mean (SD)	F/t (dfh, dfe)	*p*-Value	Effect Size η^2^*p*
P1	Within-Subjects	Electrode	-		0.157 (2, 48)	0.855	0.006
	Between-Subjects	Group (CI vs. NH)	-	-	2.681 (2, 48)	0.079	0.100
	Interaction	Electrode × Group	-	-	2.056 (1, 48)	0.158	0.040
	Pairwise Comparisons	CI vs. NH at O1	7.65 (4.22)	10.60 (4.75)	-	0.035 *	-
		CI vs. NH at O2	8.82 (4.18)	9.47 (4.17)	-	0.755	-
		CI vs. NH at OZ	8.65 (4.02)	10.73 (5.53)	-	0.133	-
NI	Within-Subjects	Electrode	-	-	0.00 (2, 50)	0.99	0.000
	Between-Subjects	Group (CI vs. NH)	-	-	0.48 (2, 50)	0.618	0.010
	Interaction	Electrode × Group	-	-	1.74 (1, 50)	0.193	0.034
	Pairwise Comparisons	CI vs. NH at O1	4.18 (2.38)	4.85 (2.23)	-	0.301	-
		CI vs. NH at O2	4.18 (2.65)	4.63 (2.16)	-	0.338	-
		CI vs. NH at O2	3.78 (2.33)	4.81 (2.27)	-	0.112	-

* *p* < 0.05.

**Table 3:** Comparison of P1 and N1 latency between cochlear implant (CI) and normal-hearing (NH) groups: results of mixed ANOVA including main effects, interaction, and pairwise analyses across electrode sites (O1, O2, and Oz).

Latency (ms)	Effect Type	Source	CI (*n* = 25) Mean (SD)	NH (*n* = 28) Mean (SD)	F/t (dfh, dfe)	*p*-Value	Effect Size η^2^*p*
P1	Within-Subjects	Electrode	-		0.00 (2, 50)	0.996	0.00
	Between-Subjects	Group (CI vs. NH)	-		0.08 (1, 50)	0.771	0.002
	Interaction	Electrode × Group	-		1.23 (2, 50)	0.295	0.024
	Pairwise Comparisons	CI vs. NH at O1	147.66 (24.69)	148.44 (24.35)	-	0.91	-
		CI vs. NH at O2	142.34 (19.74)	150.46 (26.01)	-	0.38	-
		CI vs. NH at OZ	141.72 (20.60)	139.72 (18.96)	-	0.7	-
NI	Within-Subjects	Electrode	-	-	0.020 (2, 49)	0.965	0.001
	Between-Subjects	Group (CI vs. NH)	-	-	0.590 (1, 50)	0.446	0.012
	Interaction	Electrode × Group	-	-	4.169 (2, 49)	0.021 *	0.146
	Pairwise Comparisons	CI vs. NH at O1	284.92 (29.16)	262.56 (33.51)	-	0.025 *	-
		CI vs. NH at O2	287.27 (30.83)	273.37 (27.00)	-	0.129	-
		CI vs. NH at O2	277.67 (24.15)	275.11 (23.44)	-	0.700	-

* *p* < 0.05.

## Data Availability

The original contributions presented in the study are included in the article; further inquiries can be directed at the corresponding authors.

## Supplementary Materials

The following supporting information can be downloaded at https://www.mdpi.com/article/10.3390/children12030278/s1, Figure S1: High contrast sinusoidal concentric grating (0.8 c/deg), subtending 10 deg², followed 600 ms after onset by a similar grating radially modulated in frequency [23,26]; Table S1: Demographic and Clinical Characteristics of Study Participants with Cochlear Implants (CI); Figure S2: Grand average visual evoked potentials (VEPs) recorded from occipital ROI comparing normal hearing (NH, red trace) and cochlear implant (CI, blue trace) groups. Two major components are highlighted: P1 and N1. The time course spans from −100 pre-stimulus to 400 ms post-stimulus onset, with amplitude measured in microvolts (μV); Table S2: Associations Between Demographic Variables and VEP Component Parameters (P1, N1) in the Occipital ROI.
