# Supplementary material for: Visual Cortical Processing in Children with Early Bilateral Cochlear Implants: A VEP Analysis
Source: Children (Basel). 2025 Feb 25;12(3):278. doi: 10.3390/children12030278 (PMC11940883; doi:10.3390/children12030278)
Supplement: Supplementary file 1 [file children-12-00278-s001.zip › children-3470712-supplementary.pdf]

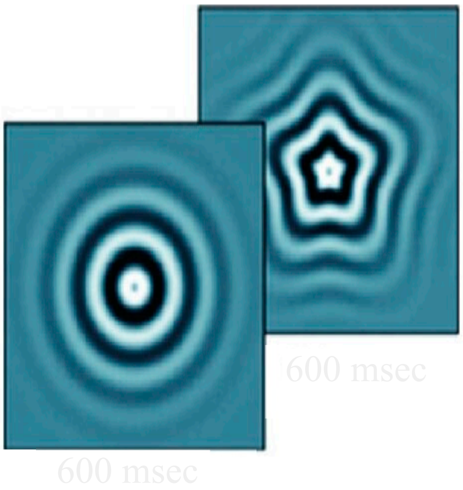


Figure S1: High contrast sinusoidal concentric grating (0.8 c/deg), subtending 10 deg2, followed 600 msec after onset by a similar grating radially modulated in frequency (Doucet et al, 2006; Campbell and Sharma, 2016)

Table S1: Demographic and Clinical Characteristics of Study Participants with Cochlear Implants (CI)

| Subject | Gender | Age at Testing (Years) | Age at 1st CI (Years) | Age at 2nd CI Years) | Implant use duration | Implant (Processor) | Etiology |
|---|---|---|---|---|---|---|---|
| CI1 | M | 8.94 | 2.94 | 2.94 | 6 | Medel (Sonnet 2) | Unknown |
| CI2 | M | 8.01 | 1.02 | 1.02 | 7 | Cochlear Nucleus 7 (CP1000) | Unknown |
| CI3 | F | 10.63 | 0.97 | 0.97 | 9.67 | Cochlear Nucleus 7 (CP1000) | Familial |
| CI4 | M | 7.94 | 2.47 | 2.47 | 5.48 | Medel (Sonnet 2) | Unknown |
| CI5 | M | 12.87 | 0.94 | 3.02 | 11.94 | Cochlear Nucleus 7 (CP1000) | Familial |
| CI6 | M | 7.9 | 0.96 | 0.96 | 6.95 | Cochlear Nucleus 7 (CP1000) | Familial |
| CI7 | M | 7.04 | 1.01 | 1.01 | 6.04 | Advanced Bionec (M90) | Familial |
| CI8 | M | 7.95 | 1.24 | 1.24 | 6.72 | Cochlear Nucleus 7 (CP1000) | Familial |
| CI9 | F | 13.23 | 1.07 | 1.92 | 12.19 | Cochlear | Unknown |

| | | | | | | | |
|---|---|---|---|---|---|---|---|
| | | | | | | Nucleus 7 (CP1000) | |
| CI10 | M | 10.05 | 1.48 | 1.48 | 8.59 | Cochlear Nucleus 7 (CP1000) | Familial |
| CI11 | M | 13.12 | 0.47 | 1.09 | 12.77 | Medel (Sonnet 2) | Familial |
| CI14 | M | 8.73 | 3.01 | 3.01 | 5.84 | Cochlear Nucleus 7 (CP1000) | Unknown |
| CI15 | F | 11.97 | 0.72 | 0.72 | 10.83 | Medel (Sonnet 2) | Unknown |
| CI16 | M | 8.19 | 1.01 | 1.01 | 7.71 | Cochlear Nucleus 7 (CP1000) | Unknown |
| CI17 | M | 8.86 | 0.89 | 0.89 | 7.67 | Cochlear Nucleus 7 (CP1000) | Unknown |
| CI19 | F | 11.66 | 1.78 | 1.78 | 10.19 | Cochlear Freedom | Familial |
| CI20 | F | 9.7 | 1.07 | 1.56 | 8.3 | Cochlear Nucleus 7 (CP1000) | Suspected Meningitis |
| CI21 | F | 8.15 | 1.09 | 1.09 | 7.39 | Cochlear Nucleus 7(CP1000) | Genetic |
| CI22 | F | 11.57 | 1.43 | 1.43 | 10.15 | Cochlear Freedom | Unkown |
| CI23 | M | 12.1 | 1.71 | 1.71 | 10.4 | Cochlear Nucleus 7 (CP1000) | Familial |
| CI24 | M | 11.99 | 0.61 | 0.61 | 11.38 | Cochlear Nucleus 7 (CP1000) | Bilirubin |
| CI25 | M | 9.14 | 1.91 | 1.22 | 7.25 | Cochlear Nucleus 7 (CP1000) | Unknown |
| CI26 | F | 9.86 | 2.74 | 2.74 | 7.14 | Medel (Sonnet 2) | Unknown |
| CI27 | M | 9.93 | 2.09 | 2.09 | 7.85 | Medel (Sonnet 2) | Unknown |
| CI28 | F | 8.49 | 1.5 | 1.5 | 7 | Cochlear Nucleus 7 (CP1000) | Unknown |

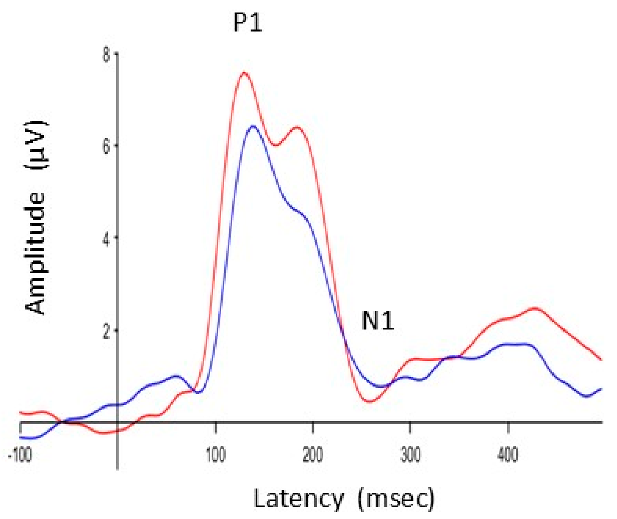


Figure S2: Grand average visual evoked potentials (VEPs) recorded from occipital ROI comparing normal hearing (NH, red trace) and cochlear implant (CI, blue trace) groups. Two major components are highlighted: P1 and N1. The time course spans from -100 pre-stimulus to 400 msec post-stimulus onset, with amplitude measured in microvolts (μV).

Table S2: Associations Between Demographic Variables and VEP Component Parameters (P1, N1) in the Occipital ROI

| | | Standardized (N1_latency) | Standardized (P1_latency) | Standardized P1 amplitude | Standardized (N1 amplitude) | Standardized (Age) | Standardized (TR_implant age ) | Standardized (Implant duration ) |
|---|---|---|---|---|---|---|---|---|
| Standardized (N1_latency) | Pearson Correlation | 1 | -.469** | .278* | .433** | -.144 | -.037 | -.203 |
| | Sig. (2-tailed) | | <.001 | .048 | .001 | .307 | .864 | .352 |
| | N | 52 | 52 | 51 | 51 | 52 | 24 | 23 |
| Standardized (P1_Latency) | Pearson Correlation | -.469** | 1 | -.070 | -.116 | -.119 | -.211 | .018 |
| | Sig. (2-tailed) | <.001 | | .620 | .412 | .398 | .312 | .933 |
| | N | 52 | 53 | 52 | 52 | 53 | 25 | 24 |

| | | | | | | | | |
|---|---|---|---|---|---|---|---|---|
| Standardized (NI amplitude) | Pearson Correlation | .278* | -.070 | 1 | .473** | -.309* | .025 | -.193 |
| | Sig. (2-tailed) | .048 | .620 | | <.001 | .026 | .905 | .366 |
| | N | 51 | 52 | 52 | 52 | 52 | 25 | 24 |
| Standardized (P1 amplitude ) | Pearson Correlation | .433** | -.116 | .473** | 1 | -.411** | .114 | -.390 |
| | Sig. (2-tailed) | .001 | .412 | <.001 | | .002 | .588 | .060 |
| | N | 51 | 52 | 52 | 52 | 52 | 25 | 24 |
| Standardized (age) | Pearson Correlation | -.144 | -.119 | -.309* | -.411** | 1 | -.320 | .945** |
| | Sig. (2-tailed) | .307 | .398 | .026 | .002 | | .119 | <.001 |
| | N | 52 | 53 | 52 | 52 | 53 | 25 | 24 |
| Standardized (TR_implant_age) | Pearson Correlation | -.037 | -.211 | .025 | .114 | -.320 | 1 | -.584** |
| | Sig. (2-tailed) | .864 | .312 | .905 | .588 | .119 | | .003 |
| | N | 24 | 25 | 25 | 25 | 25 | 25 | 24 |
| Standardized (implant duration) | Pearson Correlation | -.203 | .018 | -.193 | -.390 | .945** | -.584** | 1 |
| | Sig. (2-tailed) | .352 | .933 | .366 | .060 | <.001 | .003 | |
| | N | 23 | 24 | 24 | 24 | 24 | 24 | 24 |

** Correlation is significant at the 0.01 level (2-tailed).

* Correlation is significant at the 0.05 level (2-tailed).
